# Potassium deficiency impairs photosynthetic induction via disrupted electron transport and photochemistry in *Phaseolus vulgaris*

**DOI:** 10.3389/fpls.2026.1722785

**Published:** 2026-02-13

**Authors:** Liling Wu, Qi Luo, Ting Zhu, Yunmin Wei

**Affiliations:** 1 Nanchong Key Laboratory of Individualized Drug Therapy, Department of Pharmacy, The Second Clinical School of North Sichuan Medical College, Beijing Anzhen Nanchong Hospital of Capital Medical University & Nanchong Central Hospital, Nanchong, China; 2 Key Laboratory of Plant Genetics and Molecular Breeding, Zhoukou Normal University, Zhoukou, China

**Keywords:** electron transport rate, non-photochemical quenching, photochemical quenching, photosynthetic induction, potassium deficiency

## Abstract

Our previous study revealed that potassium (K) deficiency depressed carbon assimilation during photosynthetic induction in *Phaseolus vulgaris*. Building on this work, the current study presents a re-analysis of previously collected gas exchange and fluorescence measurements to further explore the contribution of electron transport and its components in regulating carbon assimilation under K deficiency, specifically examining electron transport rate (*J*), the fraction of open PSII reaction centers (q_L_), and non-photochemical quenching (NPQ). Our re-analysis revealed that plants supplied with deficient potassium demonstrated pronounced delay in electron transport activation, with c.430 s to achieve steady-state conditions compared to potassium sufficient plants (c. 215 s), which the delay was primarily attributed to slower q_L_ increment and the sustained NPQ elevation. Normal K supplied plants maintained higher q_L_ and *J* values throughout the induction, while NPQ levels eventually converged between treatments. Increased q_L_ led to higher photosynthesis, highlighting q_L_ as a key limiting factor in early photosynthesis induction. Taken together, under potassium deficiency, the delayed activation of electron transport reduces photosynthetic rates during induction, suggesting that improving q_L_ could enhance photosynthesis under K-deficient conditions.

## 1 Introduction

Potassium (K) is an essential element that affects most of the biochemical and physiological processes involved in plant growth and metabolism, as well as enhancing plant tolerance to abiotic stresses (e.g., drought, salinity, and extreme temperatures) (Wang and Wu, 2013; Wang et al., 2013). Importantly, it tightly regulates photosynthetic efficiency by modulating stomatal conductance, maintaining chloroplast structural integrity, as well as facilitating the synthesis and translocation of photosynthates and thereby govern plant growth and productivity (White, 2013; Rawat et al., 2022). ^K^ deficiency remains a prevalent and significant constraint on crop productivity, particularly in intensive agricultural systems, sandy or leached soils, and regions where fertilization fails to meet crop K requirements (Pettigrew, 2008; Römheld and Kirkby, 2010; Zörb et al., 2014; Liang et al., 2025). It worth noting that considerable progress has been made in elucidating the physiological mechanisms underlying impaired leaf photosynthesis under steady-state light conditions due to K deficiency (Lu et al., 2019; Galvis et al., 2020; Li et al., 2021; Yan et al., 2023). However, in natural environments, light intensity fluctuates frequently due to canopy shading, cloud cover, and solar movement (Pearcy, 1990; Tanaka et al., 2019). Under dynamic light conditions, photosynthetic induction, which refers to the time-dependent increase in photosynthesis following transitions from low to high light, is critical for cumulative carbon gain and plant productivity (Sakoda et al., 2021). Despite the well-established role of other nutrients, such as nitrogen, in regulating photosynthetic induction, how potassium affects this dynamic process remains largely unknown.

Photosynthetic induction is governed by multiple interacting factors that determine the kinetics of carbon assimilation following a transition from low to high light. Current mechanistic evidence suggests that these factors include stomatal opening, mesophyll conductance to CO_2_ (g_m_), and the activation state of enzymes in the Calvin–Benson cycle, particularly Rubisco. In addition, the dynamics of electron transport and ATP/NADPH production also play essential roles in supporting rapid induction and carbon gain (Han et al., 2022; Liu et al., 2022). The coordination of diffusional and biochemical processes during induction is crucial for maximizing photosynthetic efficiency under fluctuating light. Nevertheless, how these processes are prioritized and regulated can shift markedly in response to environmental cues and nutrient availability, reflecting a complex and context-dependent coordination (Lawson and Blatt, 2014; McAusland et al., 2016; Lazár et al., 2022; Sakoda et al., 2022).

In the context of photosynthetic induction, biochemical component is involving the activation of Calvin–Benson cycle enzymes and the dynamics of photochemical processes. Rubisco activation, RuBP regeneration, and thylakoid electron transport collectively determine the photosynthetic capacity under rising light. RuBP regeneration, in turn, depends on the supply of ATP and NADPH produced via electron transport (Sakoda et al., 2022). Two important chlorophyll fluorescence parameters reflect the regulation of these processes: photochemical quenching (q_L_), which indicates the fraction of open PSII reaction centers and photosynthetic efficiency, and non-photochemical quenching (NPQ), which dissipates excess excitation energy as heat and serves as a critical photoprotective mechanism (Han et al., 2022; Lazár et al., 2022). While the influence of stomatal and mesophyll CO_2_ diffusion and Rubisco activation on photosynthetic induction has been extensively reported (Yamori et al., 2012; Lawson and Blatt, 2014; Lazár et al., 2022; Liu et al., 2022), studies specifically addressing the role of electron transport rate in this process remain relatively scarce and often lack mechanistic depth.

Potassium deficiency further complicates these processes, as potassium plays crucial roles in maintaining enzyme activation, osmotic balance, and thylakoid membrane stability (Cui and Tcherkez, 2021; Cao et al., 2025). K regulates Rubisco activation via pH balance and Mg²^+^ transport—both essential for Rubisco activation (Cakmak, 2005). Under K deficiency, impaired Rubisco activation reduces photosynthetic efficiency under both steady and fluctuating light (Luo et al., 2025). Additionally, K deficiency disrupts ΔpH across thylakoid membranes, compromising ATP synthesis and RuBP regeneration, and potentially making electron transport a major limiting factor (Galvis et al., 2020). The balance between q_L_ and NPQ underlies efficient electron transport and photoprotection (Han et al., 2022). Potassium deficiency may shift this balance by reducing q_L_ and altering NPQ, impairing energy use and carbon assimilation during light transitions. Despite its importance, the effect of K deficiency on q_L_ and NPQ dynamics during induction is poorly understood. The relative contributions of diffusional (g_s_, g_m_) and biochemical (ETR-driven) limitations under K stress also remain unresolved.

To address these conceptual gaps and to move beyond the current focus on CO_2_ diffusion, the present study specifically examines the role of electron transport and its regulatory components during induction. For this purpose, we used common bean (*Phaseolus vulgaris*) to examine how K deficiency affects electron transport and its regulatory components, q_L_ and NPQ, during photosynthetic induction. We hypothesized that K deficiency delays the increase in electron transport by reducing q_L_ and sustaining elevated NPQ during the transition from low to high light. According to this hypothesis, we further predicted a redistribution of photochemical energy, reflected by a decline in Φ_PSII_ and a concomitant rise in Φ_NPQ_. By explicitly linking K nutrition to photosynthetic energy allocation under dynamic irradiance, this study provides mechanistic insight into K-mediated regulation of dynamic photosynthesis, offering insight into K-mediated physiological responses and informing breeding and nutrient strategies for improved photosynthetic resilience under fluctuating light.

## 2 Materials and methods

### 2.1 Plant material

The test cultivar used in this experiment was “Henan Roudou” (a local kidney bean cultivar in Henan Province), supplied by Henan Huaxia Seed Industry Co., Ltd. The cultivation procedure was identical to that described in our previous study (Yamori et al., 2012). Briefly, a hydroponic culture experiment was conducted in a controlled plant growth room with an illuminated light source providing ~800 μmol m^-2^ s^-1^ photosynthetic photon flux density (PPFD). The photoperiod was set to 16 h at 25 °C (day) and 8 h at 18 °C (night), and relative humidity was maintained at 50–60%. *P. vulgaris* seeds were germinated on moist filter paper at 4 °C for 12 h and then transferred to a transit box for germination.

After 7 d, uniform seedlings were transplanted into 10 L containers with one-quarter-strength nutrient solution. Five days later, the solution was replaced with half-strength, and after another 5 d, full-strength solutions were applied with either K-deficient (−K, 0.02 mM K_2_SO_4_) or normal K (+K, 2 mM K_2_SO_4_) treatments. Full-strength nutrient solution was applied with K treatments as described in our previous study (Luo et al., 2025). The pH was adjusted daily to 6.0 ± 0.05 for all solution strengths/treatments and the nutrient solution was renewed every 3 d.

### 2.2 Measurement of gas exchange and fluorescence during photosynthetic induction

Gas exchange and chlorophyll fluorescence data used in this study were obtained from our previous work (Luo et al., 2025), in which fully expanded leaves (five replicates for each treatment) were sampled two weeks after treatment initiation. Measurements were divided into two parts. The first set was conducted between 9:00 and 11:30 using a portable gas exchange system (Li-6400XT, Li-Cor, Lincoln, NE, USA) under the following conditions: vapor pressure deficit (VPD) of 1.4–1.6 kPa, an air flow rate of 500 μmol s^-1^, a leaf temperature of 25 ± 0.3 °C, and a reference CO_2_ concentration of 400 μmol mol^-1^. Based on our previously established protocol, leaves were initially acclimated to steady-state conditions under a low light (100 μmol m^-2^ s^-1^ photosynthetic photon flux density, PPFD) for 30 min, followed by exposure to high light (1000 μmol m^-2^ s^-1^ PPFD). Gas exchange parameters—including net photosynthetic rate (*A*), intercellular CO_2_ concentration (C_i_), and stomatal conductance to water vapor (g_s_)—were recorded every second for the first minute and every 5 seconds thereafter until *A* and g_s_ stabilized. Plants were randomly selected and measured within the 9:00–11:30 time window.

To measure F_s_ and F_m_’, all data were collected using the LI-6400 photosynthesis system (Li-Cor Biosciences, Lincoln, NB, USA) equipped with the leaf chamber fluorometer (Li-Cor Part No. 6400-40, 2 cm² chamber area). To ensure accurate estimation of F_m_’, the multi-phase flash (MPF) protocol was employed. The MPF settings were optimized with the following parameters: flash intensity of 8500 µmol m^-2^ s^-1^, measuring beam intensity of 1–2 µmol m^-2^ s^-1^, a 60% reduction in flash intensity during the second phase, and durations of 0.3, 0.7, and 0.4 seconds for the three successive flash phases. These fluorescence parameters (F_s_ and F_m_’) were logged with a time interval of 74–78 s. The second part of the measurement involved determining minimal (F_o_) and maximal (F_m_) fluorescence. Plants were dark-adapted overnight and measured with a rectangular-type saturating flash between 6:00 and 7:00. Leaf chamber conditions were identical to those used in the first part, except that light intensity was set to zero. The F_o_/F_o_′, F_m_/F_m_′, and F_s_ time series are shown in **Supplementary Figure S2**.

### 2.3 Calculations of chlorophyll fluorescence parameters

The maximum quantum efficiency of photosystem II was calculated using fluorescence measurements from dark-adapted leaves:

(1) $$\begin{array}{c} \Phi_{PSII\text{max}}=\frac{F_{m}-F_{o}}{F_{m}} \end{array}$$

The fraction of open reaction centers in PSII (q_L_) during photosynthetic induction can be derived by:

(2) qL=Fm'-FsFm'-Fo'×Fo'Fs

where F_o_′ denotes the minimum fluorescence yield under light-adapted conditions, commonly measured by using far-red illumination to facilitate electron withdrawal and achieve full oxidation of the primary quinone acceptor of photosystem II. As noted by Han et al (Han et al., 2022), F_o_′ is often overestimated during direct measurements. Therefore, a calculated value was used instead, based on the following equation (Baker and Oxborough, 2004):

(3) $$F_{o}^{\prime }=\frac{F_{o}}{\Phi_{PSII\text{max}}+\frac{F_{o}}{F_{m}^{\prime }}}$$

Non-photochemical quenching (NPQ) can be calculated as follows:

(4) $$NPQ=\frac{F_{m}-F_{m}^{\prime }}{F_{m}^{\prime }}$$

To assess the quantum efficiencies of distinct pathways involved in the utilization of absorbed light energy during the photosynthetic induction period, we calculated the actual photochemical efficiency of photosystem II (Φ_PSII_), the quantum yield of regulated thermal energy dissipation via non-photochemical quenching (Φ_NPQ_), and the quantum yield of non-regulated energy dissipation through additional quenching mechanisms (Φ_f, D_) following Hendrickson et al (Hendrickson et al., 2004):

(5) $$\Phi PSII=\frac{F_{m}^{\prime }-F_{s}}{F_{m}^{\prime }}$$

(6) $$\Phi NPQ=\frac{F_{s}}{F_{m}^{\prime }}-\frac{F_{s}}{F_{m}}$$

(7) $$\begin{array}{c} \Phi_{f,\;D}=\frac{F_{s}}{F_{m}} \end{array}$$

The calculation of linear electron transport rate (*J*) was performed using the equation below:

(8) $$\begin{array}{c} J=\Phi_{PSII}\times \alpha \times \beta \times PPFD \end{array}$$

Here, α represents leaf absorbance, and β denotes the proportion of absorbed light directed to photosystem II, as described by Yin et al (Yin et al., 2009). Following their method, we estimated the combined parameter αβ by establishing a linear relationship between Φ_PSII_ × PPFD/4 and *A*, based on data from a typical steady-state light response curve measured under 2% O_2_ conditions. The fitted αβ values were as follows: for the K-deficient leaves, αβ = 0.4880 ± 0.0189, and for the +K treatment, αβ = 0.4893 ± 0.0160. No significant difference was observed between the two treatments. The corresponding plot for this relationship is presented in **Supplementary Figure S1**, with the fitted R² values exceeding 0.999 in all cases.

The photosynthetic induction state (IS) was quantified using the following equations, in which the prefixes ‘i’ and ‘f’ indicate measured chlorophyll parameters (X; i.e. ΦPSII, ΦNPQ, Φf, D, qL, NPQ, J) during the final minute under low light and high light conditions, respectively:

(9) $$\begin{array}{c} IS=\frac{X\left(t\right)-iX}{fX-iX} \end{array}$$

where X(t) represents chlorophyll parameters at any time point during the induction period. Additionally, t_J90_ was defined as the time required for *J* to reach 90% of the difference between i*J* and f*J*, and similarly, t_qL90_ and t_NPQ90_ were defined as the times required for q_L_ and NPQ to reach 90% of the differences between iq_L_ and fq_L_, and iNPQ and fNPQ, respectively.

### 2.4 Assessment of the relative impact of qL and NPQ on photosynthetic performance

To evaluate the individual contributions of qL and NPQ to carbon gain during induction, we substituted all dynamic values of either qL or NPQ during the photosynthetic induction process with their respective fixed values at the end of high light period—designated as fqL or fNPQ. These fixed values were then used to recalculate J during light transition as below following Gu et al. (2019):

(10) $$\begin{array}{c} J=\frac{q_{L}\times \Phi_{PSII\text{max}}}{\left(1+NPQ\right)\times \left(1-\Phi_{PSII\text{max}}\right)+q_{L}\times \Phi_{PSII\text{max}}}\times \alpha \times \beta \times PPFD \end{array}$$

Accordingly, A was recalculated using the following Equation:

(11) $$\begin{array}{c} A=\frac{J\times \left(C_{c}-\Gamma^{*}\right)}{4C_{c}+8\Gamma^{*}}-R_{d} \end{array}$$

where Rd corresponds to the rate of respiration occurring in the light; Cumulative carbon gain (C gain, mmol m-2) was calculated by integrating the A over the light induction period, using the measured or recalculated values for −K treatment. The total time was 995 s, with a time interval of 74–78 s. Since the fluorescence parameters (Fs and Fm’) used for recalculating qL and NPQ were logged with a time interval of 74–78 s, the recalculated A values and the integration for C gain were also performed using this time interval. Cc, representing the CO_2_ concentration within the chloroplast, was calculated as follows:

(12) $$\begin{array}{c} C_{c}=\frac{\Gamma^{*}\times J+8\times A+R_{d}}{J-4\times A+R_{d}} \end{array}$$

G* indicates the CO_2_ compensation point without the influence of mitochondrial respiration, which can be derived by:

(13) $$\begin{array}{c} \Gamma^{*}=C_{i}^{*}+\frac{R_{d}}{g_{m}} \end{array}$$

where C_i_^*^ represents the apparent CO_2_ photocompensation point. R_d_ and C_i_^*^ were estimated using the method described by Brooks et al (Brooks and Farquhar, 1985), in which *A*/C_i_ response curves were measured under three sub-saturating photosynthetic photon flux densities (150, 300, and 600 μmol m^-2^ s^-1^). The intersection points of these curves provided the x- and y-intercepts, corresponding to C_i_^*^ and R_d_, respectively. The two remaining unknowns in Eqs. (12) and (13)—C_c_ and Γ^*^—were subsequently resolved by simultaneous solution of the two equations.

### 2.5 Data provenance and new analyses

The data presented in this study include a combination of previously published gas-exchange and fluorescence measurements. Specifically, the raw variables used in this study include *A*, g_s_, F_s_, F_m_′, and F_m_, as well as *J* and g_m_. These raw variables were obtained from our prior work and serve as the foundation for the analyses presented here. In addition to the reuse of these raw measurements, several newly computed derived variables and analyses are introduced in this manuscript. These new variables include: (i) q_L_, (ii) NPQ, (iii) the partitioning of absorbed light between photosystem II and non-photochemical quenching (Φ_PSII_/Φ_NPQ_/Φ_f,D_), and (iv) the fixed-qL/NPQ *A* approach to estimate photosynthetic efficiency under fluctuating light. These new contributions provide fresh insights into the photosynthetic induction process and extend the interpretation of the previous data.

### 2.6 Statistical analysis

To evaluate the effects of different potassium treatments on physiological parameters, Student’s *t*-tests was performed (*P* < 0.05), using SPSS software (version 20.0; SPSS Inc., Chicago, IL, USA). The C gain under fixed q_L_ was compared with that under fixed NPQ and measured *A* using paired t-tests in the potassium deficiency treatment.

## 3 Results

### 3.1 Effects of potassium on photochemical parameters during light induction

**Figure 1:**
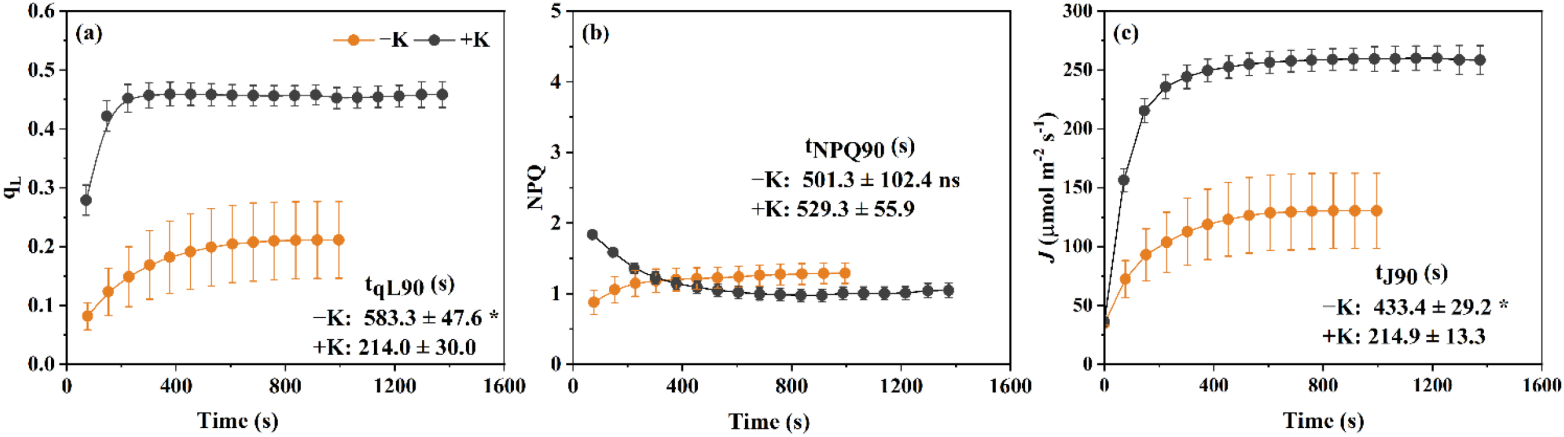
Temporal responses of photosynthetic parameters to light induction in *Phaseolus vulgaris* under potassium-deficient (−K) and potassium-sufficient (+K) treatments. **(a)** q_L_, the fraction of open PSII reaction centers; **(b)** NPQ, non-photochemical quenching; **(c)** J, electron transport rate. The times required to reach steady-state values of q_L_, NPQ, and J are indicated for both −K and +K treatments. Data are presented as means ± SE (n = 5). Asterisks indicate significant differences in t_J90_ and t_qL90_ between the −K and +K treatments (*p* < 0.05); ns indicates no significant difference.

To investigate the effect of potassium availability on photochemical performance in *Phaseolus vulgaris*, we monitored the temporal responses of q_L_, NPQ, and *J* under potassium-deficient (−K) and potassium-sufficient (+K) conditions during light induction (**Figure 1**). Under +K treatment, q_L_ increased rapidly after light exposure, and the time required for qL to achieve 90% of the initial to final value difference was 214.0 ± 30.0 s. In contrast, under −K conditions, plants displayed markedly slower q_L_ kinetics, rising more gradually and the time required for qL to reach 90% of the initial to final value difference was 583.3 ± 47.6 s (**Figure 1a**), indicating delayed oxidation of Q_A_ (the primary plastoquinone electron acceptor of PSII) and impaired electron transport activation.

Inversely, NPQ exhibited divergent trends between treatments. While NPQ in +K plants decreased steadily from ~1.8 to ~1.0 over 25 minutes, −K plants showed a gradual increase in NPQ during the induction phase (**Figure 1b**), reflecting enhanced non-photochemical quenching under potassium deficiency. The time required for NPQ to reach 90% of the initial to final value difference was 501.3 ± 102.4 s in −K plants and 529.3 ± 55.9 s in +K plants, although this difference was not statistically significant. Although *J* increased over time in both treatments, its activation was faster under sufficient potassium supply. The time required to approach 90% of the initial to final value difference for *J* was significantly shorter in +K plants (214.9 ± 13.3 s) compared to −K plants (433.4 ± 29.2 s; **Figure 1c**), suggesting that K facilitates a more efficient activation of electron transport.

**Figure 2:**
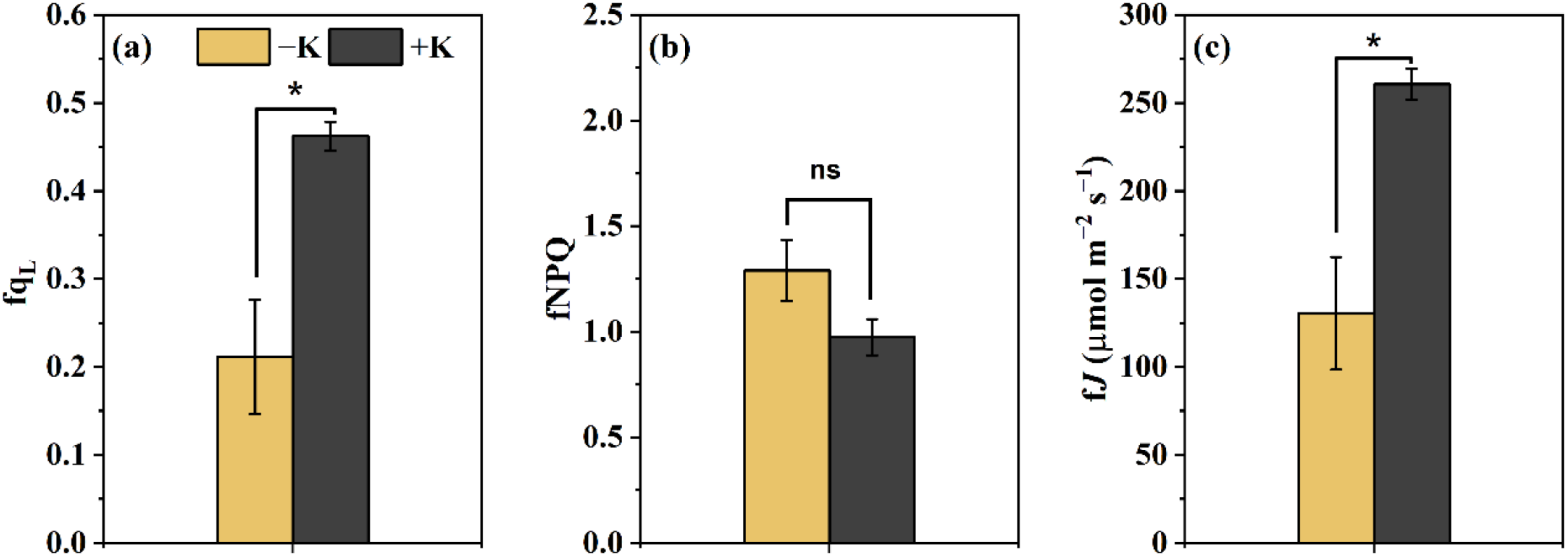
Steady-state values of photosynthetic parameters measured at the end of the light induction phase in *Phaseolus vulgaris* under potassium-deficient (−K) and potassium-sufficient (+K) treatments. **(a)** q_L_, the fraction of open PSII reaction centers; **(b)** NPQ, non-photochemical quenching; **(c)** *J*, electron transport rate. Data are presented as means ± SE (n = 5). Asterisks indicate denote significant differences between the two treatments for fqL and f*J* (*p* < 0.05); ns indicates no significant difference.

Consistent with these trends, the steady-state levels of q_L_ and *J* were significantly higher under +K conditions than under −K (**Figures 2a, c**). Interestingly, no significant difference in steady-state NPQ was observed between treatments (**Figure 2b**), indicating that the prolonged NPQ observed under −K may primarily affect the dynamic phase rather than steady-state regulation.

### 3.2 Dynamic effects of photochemical parameters on photosynthetic induction

**Figure 3:**
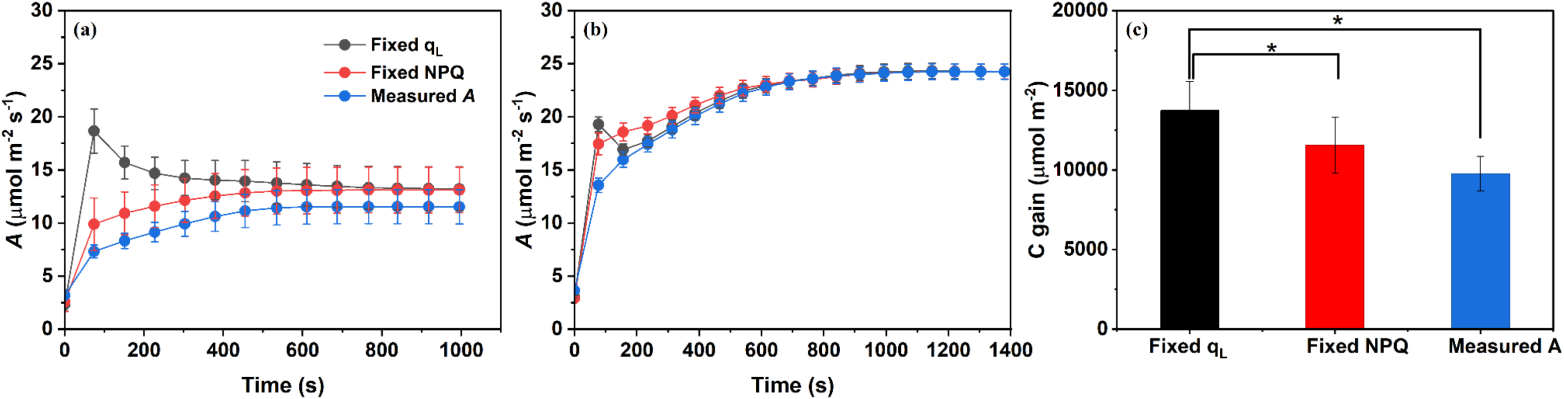
Re-estimated net CO_2_ assimilation rate (A) by fixing q_L_ and NPQ to their steady-state values following light induction in Phaseolus vulgaris under potassium-deficient [−K; **(a)**] and potassium-sufficient [+K; **(b)**] treatments, together with the cumulative carbon gain (C gain) derived from measured and recalculated *A* under −K treatment **(c).** *A* was recalculated as described in Materials and Methods by fixing either q_L_ or NPQ to their respective steady-state values attained at the end of the light induction phase, and applying these fixed values across the entire induction period. q_L_ represents the fraction of open PSII reaction centers and indicates the redox state of Q_A_ (the primary quinone electron acceptor of PSII), while NPQ (non-photochemical quenching) reflects the thermal dissipation of excess excitation energy. Data are presented as means ± SE (n = 5).

To assess the contribution of q_L_ and NPQ to CO_2_ assimilation during light induction, we fixed the measured q_L_ and NPQ values at each time point to their steady-state values at the ends of induction, and applied these values across all time points to recalculated *A* under both treatments (**Figures 3a, b**). Specifically, in the −K condition, fixing q_L_ led to a substantial overestimation of *A* in the early stages of induction, with predicted *A* peaking almost immediately after light exposure and then gradually declining toward the measured steady-state value.

Specifically, cumulative C gain during induction was 13720.9 ± 1839.0 μmol m^-2^ for the fixed q_L_ scenario, 11556.3 ± 1758.7 μmol m^-2^ for the fixed NPQ scenario, and 9761.3 ± 1084.4 μmol m^-2^ for the measured *A* under potassium deficiency. Statistical analysis revealed that C gain under fixed q_L_ was significantly higher than both fixed NPQ and measured *A* (**Figure 3c**). This discrepancy highlights the strong constraint imposed by delayed q_L_ activation under potassium deficiency. In contrast, the pattern of recalculated *A* based on fixed q_L_ closely matched the measured *A* under +K conditions. By comparison, fixing NPQ had only a minor effect on the recalculated *A* in either treatment. Although estimated *A* in −K plants was slightly higher than measured *A*, the overall shape of the photosynthetic induction curve was largely unchanged, indicating that NPQ dynamics play only a minor role in constraining *A* (**Figure 3**). Together, these results indicate that the variation in q_L_ is the primary photochemical factor limiting photosynthetic induction under K deficiency, particularly during the initial stages of light exposure.

**Figure 4:**
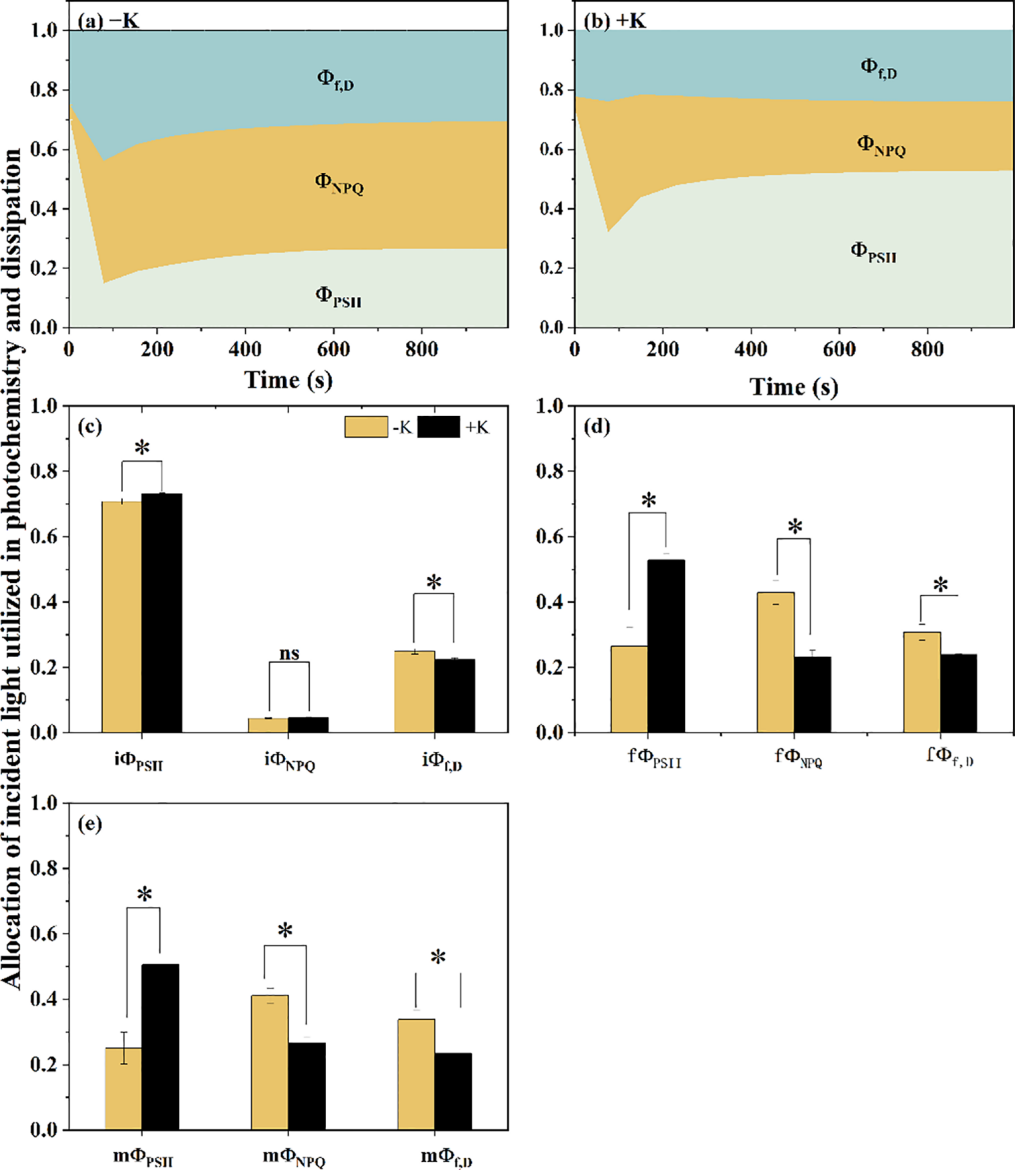
Photosynthetic energy partitioning parameters in *Phaseolus vulgaris* under potassium-deficient (−K) and potassium-sufficient (+K) treatments during the light induction period. **(a)** Energy partitioning parameters under −K treatment: Φ_PSII_ (actual photochemical efficiency of photosystem II), Φ_NPQ_ (fraction of absorbed light energy dissipated as heat via non-photochemical quenching), Φ_f_, _D_ (fraction of absorbed light energy dissipated by additional quenching mechanism). **(b) ** Φ_PSII_, Φ_NPQ_, Φ_f_, _D_ for the +K treatment. **(c) ** Initial values of energy partitioning parameters at the start of light induction (iΦ_PSII_, iΦ_NPQ_, and iΦ_f_, _D_) for both treatments; **(d) ** Final values of energy partitioning parameters at the end of induction (fΦPSII, fΦ_NPQ_, and fΦ_f_, _D_) for both treatments; **(e) ** Mean values of energy partitioning parameters during the induction period (mΦ_PSII_, mΦ_NPQ_, and mΦ_f_, _D_) for both treatments. Data are presented as means ± SE (n = 5). Asterisks indicate significant differences between treatments (*p* < 0.05); ns indicates no significant difference.

To further evaluate how incident light energy was allocated among photochemical and non-photochemical pathways during induction, we calculated the temporal changes in Φ_PDII_, Φ_NPQ_, and Φ_f, D_ (**Figures 4a, b**). In both treatments, Φ_PDII_ initially declined and then gradually increased to steady state. Φ_NPQ_, by contrast, increased sharply within the first 2 minutes, especially under −K conditions, and then plateaued or slightly declined. Φ_f, D_ also responded to light induction, though its changes were less pronounced. Notably, Φ_PSII_ was significantly higher under +K than −K throughout the induction and at steady state, indicating a greater proportion of absorbed light used in photochemistry under sufficient potassium. Conversely, Φ_NPQ_ dominated the energy partitioning at steady state in −K plants, further supporting the idea that potassium deficiency promotes thermal dissipation over photochemical utilization. The fΦ_PSII_ for the −K treatment is 0.2055 ± 0.04896, compared to 0.50496 ± 0.01864 for the +K treatment. The fΦ_NPQ_ is 0.41091 ± 0.02262 for −K and 0.26614 ± 0.01708 for +K. The fΦ_f,D_ is 0.3377 ± 0.0278 for −K and 0.23428 ± 0.00201 for +K (**Figures 4c, d**). Our results show that Φ_PSII_ is significantly higher in the +K treatment, while Φ_NPQ_ and Φ_f,D_ are significantly lower.

## 4 Discussion

### 4.1 Electron transport limitation dominates photosynthetic induction under potassium deficiency

In natural ecosystems, light conditions fluctuate frequently, and such changes can impair the photosynthetic apparatus and reduce carbon gain if plants fail to respond effectively (Eberhard et al., 2008; Pearcy and Way, 2012). This study demonstrates that potassium deficiency significantly impairs the induction of photosynthesis under fluctuating light, primarily by delaying the activation of the electron transport rate (*J*) and the redox state of PSII (q_L_), while enhancing sustained non-photochemical quenching (NPQ). These alterations reduce the efficiency of light energy utilization and limit carbon assimilation during the light transition phase.

Studies have shown that potassium deficiency reduces the efficiency of electron transport in leaves under steady-state light conditions, reflecting a limitation in photosystem II activity (Wang et al., 2015; Du et al., 2019; Lu et al., 2019). Here, we observed that the steady-state *J* decreased at the end of the induction under potassium starvation compared to normal K (**Figure 2**), and its induction rate was markedly slower, far exceeding the commonly cited 1–2 minutes required to reach near-maximal rates (Wykoff et al., 1998; Taylor and Long, 2017).

### 4.2 q_L_, Not NPQ, governs the photochemical limitation during photosynthetic induction under potassium stress

To better understand the mechanisms underlying the electron transport limitation during photosynthetic induction under potassium deficiency, we analyzed the induction-phase dynamics of q_L_ and NPQ, which represent the redox state of PSII and the non-photochemical dissipation of excitation energy, respectively. Under K deficiency, q_L_ remained substantially lower state throughout the induction phase compared to the K-sufficient condition (**Figure 1b**), indicating a persistent over-reduction of Q_A_ and a delayed activation process of electron transport (Davis et al., 2017; Kono et al., 2017). In contrast, instead of decreasing during the transition from low to high light under K-sufficient condition, NPQ continued to rise under high light relative to its value under low light under K deficiency condition (**Figure 1b**), which suggested that K deficiency significantly altered the excitation-energy allocation during induction. As a result, light energy was increasingly partitioned toward heat dissipation, reducing the energy available for photochemistry. Consistent with this, our partitioning analysis showed a significant decrease in Φ_PSII_ and increase in Φ_NPQ_ in condition of K deficiency, suggesting a shift from photochemistry toward heat dissipation under K deficient conditions (Zhu et al., 2004; Nosalewicz et al., 2022).

K is known as core factor maintaining ion balance inside and outside the thylakoid membrane. K deficiency directly inhibits the activity of K^+^/H^+^ antiporters on the thylakoid membrane (Che et al., 2022), and lead to a significant increase in proton gradient (ΔpH) (Kroll and Booth, 1981). As a result, excessively high ΔpH accelerates the activation of violaxanthin de-epoxidase (Kramer et al., 2003), promoting the conversion of violaxanthin to zeaxanthin, which can bind to light-harvesting complex II to enhance its heat dissipation capacity, ultimately and enhancing NPQ formation via the xanthophyll cycle (Demmig and Winter, 1988), which is manifested as the characteristic of persistently high Φ_NPQ_. However, the lack of key intermediate indicators, such as zeaxanthin content and ATP synthase activity may introduce certain conjecture in the interpretation of the mechanism. Further studies are needed regarding these targets to fully resolve the mechanistic pathway linking K deficiency to altered energy allocation and delayed electron transport.

Notably, the slower induction of q_L_ showed a tighter correspondence with the limitation in CO_2_ assimilation than NPQ dynamics. When we simulated *A* during induction by fixing either q_L_ or NPQ at their steady-state values at the end of high light induction, the deviation between predicted and measured *A* was substantially larger when q_L_ was fixed (**Figure 4a**). This indicates that the delay in q_L_ recovery contributed more directly to the restriction of photosynthetic induction than NPQ relaxation, in line with Han et al., who emphasized the dominant role of q_L_ in regulating *J* limitation (Han et al., 2022).

Taken together, these findings suggest that under K deficiency, the imbalance between photochemical and non-photochemical energy use is exacerbated by both sustained thermal dissipation and a prolonged over-reduction of PSII acceptors. However, it is the impaired q_L_ recovery that plays a more pivotal role in constraining the induction of photosynthesis. This underscores the importance of nutrient-dependent regulation of PSII redox balance during transitions in light availability.

## 5 Conclusions

Potassium deficiency disrupts the coordination of light energy use and carbon assimilation during photosynthetic induction under fluctuating light. This disruption manifests through delayed activation of q_L_ and *J*, enhanced NPQ, ultimately constraining carbon gain. These findings highlight the importance of sufficient K nutrition in maintaining dynamic photosynthetic efficiency and provide mechanistic insights into how nutrient stress intersects with light fluctuation responses. Compared to adjustments in NPQ, increased q_L_ enhances photosynthesis, positioning q_L_ as a key limiting factor in early photosynthetic induction. This suggests that improving q_L_ may be a more effective strategy for enhancing photosynthesis under potassium-deficient conditions. Future research should further investigate the regulatory mechanisms of q_L_ during photosynthetic induction.

## Data Availability

The raw data supporting the conclusions of this article will be made available by the authors, without undue reservation.
