# Supplementary material for: Potassium deficiency impairs photosynthetic induction via disrupted electron transport and photochemistry in Phaseolus vulgaris
Source: Front Plant Sci. 2026 Feb 13;17:1722785. doi: 10.3389/fpls.2026.1722785 (PMC12946011; doi:10.3389/fpls.2026.1722785)
Supplement: Supplementary file 1 [file DataSheet1.docx]

Supplemental material

**
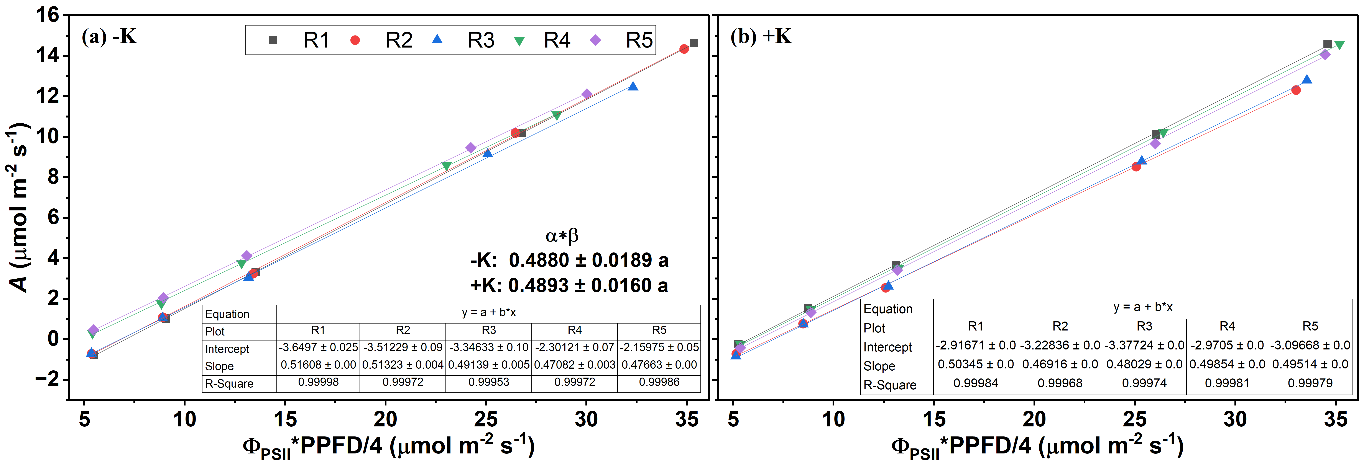
**

**Fig. S1.** The ΦPSII×PPFD/4 vs *A* relationship for -K and +K treatments. Its slope is α*β, with data presented as mean ± SE, n=5. α represents leaf absorbance, and β denotes the proportion of absorbed light directed to photosystem II. Identical letters indicate no significant difference in α*β between the two treatments.


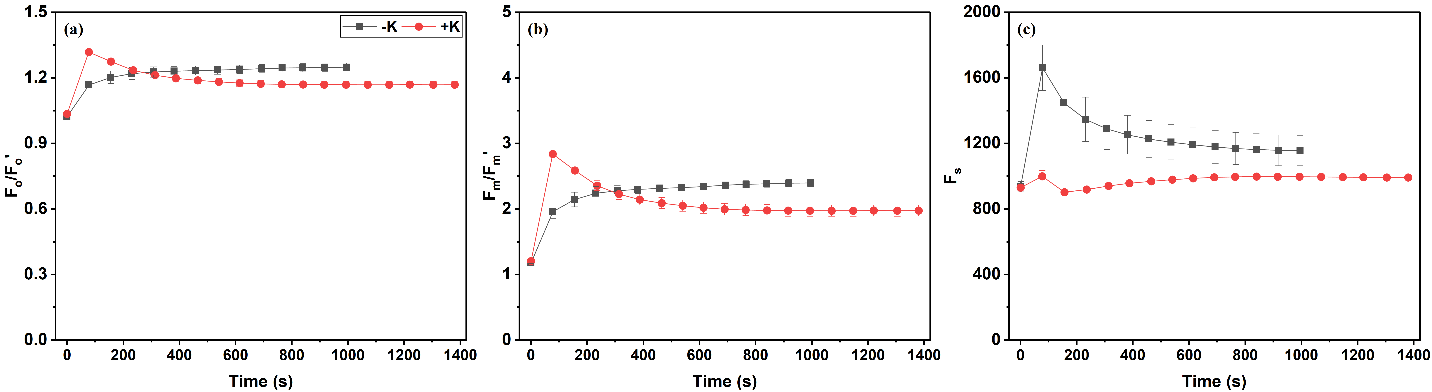


**Fig. S2.** Changes in F₀/F₀′, F_m_/F_m_′, and F_s_ during photosynthetic induction under -K and +K Treatments. Data are presented as mean ± SE, n = 5. F_o_ denotes the minimum fluorescence yield measured in dark-adapted leaves, whereas F_o_′ represents the minimum fluorescence yield under light-adapted conditions. F_m_ is the maximum fluorescence yield obtained during a saturating pulse in dark-adapted leaves, while F_m_′ is the maximum fluorescence yield measured under light-adapted conditions. F_s_ refers to the steady-state fluorescence yield under actinic light.
